# Development of machine learning models for predicting early pregnancy outcomes based on β-hCG, progesterone, and estradiol

**DOI:** 10.1371/journal.pone.0348114

**Published:** 2026-04-27

**Authors:** Luming Chen, Fangxiang Mu, Kexin Wang, Fang Wang

**Affiliations:** 1 Department of Obstetrics and Gynecology, Longxi County Traditional Chinese Medicine Hospital, Dingxi, China; 2 Department of Reproductive Medicine, Lanzhou University Second Hospital, Lanzhou, China; National Institute of Child Health and Human Development (NICHD), NIH, UNITED STATES OF AMERICA

## Abstract

**Objective:**

This study aims to develop a machine learning model for predicting early pregnancy outcomes by combining baseline levels and dynamic changes of β-human chorionic gonadotropin (β-hCG), progesterone (P), and estradiol (E2).

**Methods:**

This retrospective study screened out 421 patients treated at the Lanzhou University Second Hospital between March 2023 and August 2024. Feature selection was performed using Least Absolute Shrinkage and Selection Operator (LASSO) and Random Forest Recursive Feature Elimination (RF-RFE). Subsequently, we constructed a traditional logistic regression model and five machine learning models: Random Forest (RF), eXtreme Gradient Boosting (XGBoost), k-Nearest Neighbors (KNN), Multilayer Perceptron (MLP) neural network, and Support Vector Machine (SVM). Internal validity was assessed through 5-fold cross-validation. Model performance was measured by the area under the Receiver Operating Characteristic curve (AUC), accuracy, precision, sensitivity, and specificity.

**Results:**

Among the 421 enrolled patients, 263 had ongoing pregnancies while 158 experienced early pregnancy loss (EPL). LR, RF, XGBoost, KNN, MLP, and SVM achieved AUCs of 0.750, 0.784, 0.750, 0.706, 0.755, and 0.749, respectively, with all accuracy and precision metrics exceeding 0.60. Notably, the RF model yielded optimal performance for EPL prediction, attaining the highest AUC (0.784), accuracy (0.729), and precision (0.724).

**Conclusion:**

Integrating dynamic changes in β-hCG, P, and E2 enables effective prediction of early pregnancy outcomes. The RF model exhibited optimal performance, highlighting its potential for clinical implementation as a risk stratification tool based on serial hormone monitoring.

## 1. Introduction

Early pregnancy loss (EPL) refers to a non-viable intrauterine pregnancy occurring within the first 12 weeks of gestation, characterized by either an empty gestational sac or a gestational sac containing an embryo or fetus without cardiac activity [1]. Prospective studies indicate that EPL affects 15%–20% of clinically recognized pregnancies [2]. Beyond potential physiological symptoms such as bleeding and abdominal pain, EPL can exert profound psychological consequences on patients and their families, including grief, guilt, depression, and anxiety [3,4]. Therefore, early and accurate prediction of pregnancy outcomes is crucial for enabling timely clinical intervention and providing effective psychological support.

Currently, various biomarkers are utilized to assess early pregnancy development and predict pregnancy outcomes. β-human chorionic gonadotropin (β-hCG) is a key hormone secreted by trophoblast cells following implantation. Evidence indicates that unhealthy pregnancies exhibit slower rises or even declines in maternal serum β-hCG levels, often lacking the characteristic doubling increase [5]. Progesterone (P) is crucial for endometrial receptivity, implantation, and maintaining pregnancy [6]. Ucyigit et al. confirmed that women with lower P levels in early pregnancy suffer an increased risk of pregnancy loss [7]. Estradiol (E2) levels reflect follicular quality and corpus luteum function, contributing to luteal maintenance. Serum E2 levels are typically significantly lower in women experiencing pregnancy loss compared to those with normal pregnancies [8]. Despite their use, the clinical application of these biomarkers faces challenges. Bobdiwala et al. found that single-threshold levels for β-hCG, β-hCG ratios, and progesterone were clinically unreliable for determining early pregnancy viability [9], findings corroborated by Pillai et al. [10]. This highlights the limited predictive utility of traditional approaches relying on single-timepoint measurements or individual biomarkers.

Therefore, the development of a reliable, early-warning method for EPL is imperative to enable timely clinical intervention. This study aims to establish machine learning models for predicting early pregnancy outcomes by integrating both static and dynamic features of β-hCG, P, and E2.

## 2. Materials and methods

### 2.1. Participants

**Fig 1:**
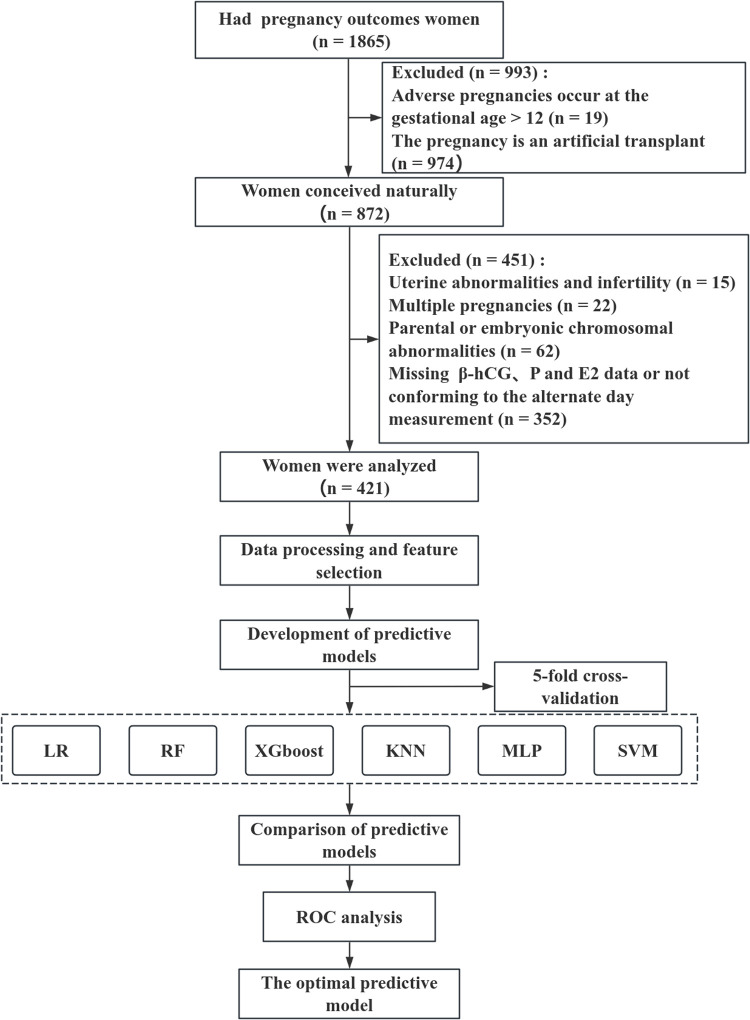
Flowchart. LR: Logistic regression; RF: Random forest; XGBoost: eXtreme Gradient Boosting; KNN: k-nearest neighbors; SVM: Support vector machine; MLP: Multilayer perceptron.

This retrospective study consecutively enrolled 1,865 patients with previous pregnancy outcomes treated at the Department of Reproductive Medicine, Lanzhou University Second Hospital, between 11 March 2023 and 26 August 2024. Inclusion criteria were as follows: (1) age between 18 and 45 years; (2) natural intrauterine conception; (3) available data on serum levels of β-hCG, P, E2, and pregnancy outcomes; (4) at least one set of alternate-day measurements of β-hCG, P, and E2 obtained within the first 12 gestational weeks; and (5) a history of pregnancy loss. The exclusion criteria were: (1) adverse pregnancy events occurred after 12 weeks of gestation; (2) chromosomal abnormalities in either parent or the embryo; (3) congenital uterine malformations (including septate, unicornuate, bicornuate, or didelphys uterus); (4) multiple gestations; (5) infertility; and (6) missing or insufficiently frequent data on β-hCG, P, or E2 levels. Ultimately, 421 patients were included in the study and categorized into the EPL group (n = 158) and the ongoing pregnancy group (n = 263), according to pregnancy outcomes at 12 weeks (Fig 1). Approval for the study was granted by the Ethics Committee at the Lanzhou University Second Hospital (Reference No. 2019A-231), and all patients provided their written informed consent.

### 2.2. Data collection

Maternal characteristics, including age, weight, height, age at menarche, regularity of menstruation, and history of pregnancy loss, were derived from medical records. Body mass index (BMI) was defined as weight divided by the square of height (kg/m²). Pregnancy outcomes were determined through follow-up or by reviewing medical records from the hospital.

### 2.3. Main outcome

We defined EPL as pregnancy loss before 12 weeks of gestation, which included biochemical pregnancies. Conversely, an ongoing pregnancy is referred to as a viable pregnancy confirmed at the 12-week gestation. In this study, the pregnancy outcomes of all the research subjects were comprehensively determined by the specialists in the Department of Reproductive Medicine, Lanzhou University Second Hospital, based on the results of ultrasound examinations, a series of serum hormone test data, and clinical symptoms. Ultrasound examinations served as the core supporting evidence for diagnosing pregnancy outcomes, providing a crucial guarantee for the accuracy of the research results.

### 2.4. Serum β-hCG, P, and E2 measurements

Hormonal assessments included initial and alternate-day β-hCG levels, P levels, and E2 levels. All measurements were performed at the Reproductive Center of Lanzhou University Second Hospital, by trained personnel using validated automated immunoassay systems. First and second log(β-hCG) refer to the log-transformed values of two β-hCG measurements taken at a 48-hour interval; β-hCG ratio refers to the ratio of the second β-hCG value to the first; interday estradiol/progesterone level difference is calculated as the difference between the second and first measurements.

### 2.5. Statistical analysis

#### 2.5.1. Data analysis.

Statistical analysis was performed using R version 4.3.1. Continuous variables were compared using the Mann-Whitney U test or t-test, while categorical variables were compared using the Chi-square or Fisher’s exact tests. Continuous variables with a normal distribution were presented as means with standard deviations (SD), while those with a skewed distribution were presented as medians with interquartile ranges (IQR). Categorical variables were shown as frequencies (percentages). Associations between variables were assessed by Pearson correlation. A two-tailed P < 0.05 is deemed statistically significant.

All statistical analyses were conducted using R software (version 4.3.1; R Core Team, 2023) within the RStudio integrated development environment (version 2023.03.0; RStudio Team, 2023). Specifically, the glmnet package (v4.1-7) was used for LASSO regression, while the caret package (v6.0-94) supported Random Forest-Recursive Feature Elimination (RF-RFE) and model training. The pROC package (v1.18.2) was utilized for receiver operating characteristic (ROC) curve analysis and area under the curve (AUC) calculation. Model construction was facilitated through randomForest (v4.7-1.1), xgboost (v1.7.6.1), and e1071 (v1.7-13) for Random Forest, Extreme Gradient Boosting, and Support Vector Machine algorithms, respectively. Additionally, the class package (v7.3-22) provided the k-Nearest Neighbors algorithm, neuralnet (v1.44.2) was used for Multilayer Perceptron training, and vip (v0.4.1) was employed to visualize variable importance.

#### 2.5.2. Feature selection.

In this research, feature selection was executed through a combination of RF-RFE and the Least Absolute Shrinkage and Selection Operator (LASSO). LASSO was implemented utilizing the *glmnet* package in R. To ensure robust model performance, the optimal regularization parameter, denoted as λ, was determined through a rigorous process of 10-fold cross-validation. In this approach, the entire dataset was randomly divided into ten distinct subsets. Each subset was utilized once as a validation set, while the remaining subsets collectively served as the training set. This design allowed for a comprehensive evaluation of various λ values during the training phase, with model performance subsequently assessed on the corresponding validation set. Ultimately, the λ that demonstrated optimal performance across all folds was selected as the final regularization parameter for the LASSO model. Simultaneously, RF-RFE was applied using the caret package in R. This technique involved a systematic approach to eliminating features, governed by specific parameters controlling the recursive elimination process. Accuracy served as the primary performance metric for this method. The iterations of feature elimination continued until either a predefined number of features was attained or optimal performance was reached. This dual approach of utilizing both LASSO and RF-RFE provided a comprehensive framework for effective feature selection, ensuring that the most pertinent features were identified for enhancing model performance.

#### 2.5.3. Model development and evaluation.

Six machine learning algorithms were developed to predict EPL, including logistic regression (LR), random forest (RF), extreme gradient boosting (XGBoost), k-nearest neighbors (KNN), multilayer perceptron (MLP), and support vector machine (SVM). Patients were randomly assigned to the training set, and 5-fold cross-validation was performed for internal validation. Model parameters were optimized through a grid search approach, with the best configuration determined after multiple iterations. Diagnostic accuracy and discriminative power were evaluated by means of ROC analysis. Evaluation metrics for each model included accuracy, precision, sensitivity, and specificity. AUC was utilized as the primary metric due to its threshold-independent nature. Conversely, threshold-dependent metrics, such as accuracy and sensitivity, were reported based on the default settings.

#### 2.5.4. Sensitivity analysis.

To evaluate the robustness of the predictive model across different populations, patients with biochemical pregnancies were excluded, and the optimal model was refitted to assess whether its predictive performance remained consistent.

## 3. Results

### 3.1. Baseline characteristics

All 421 women were enrolled in this study and underwent alternate-day measurements of serum β-hCG, P, and E2 during the first trimester (**Table 1**). Among them, 263 had ongoing pregnancies at 12 weeks, while 158 experienced EPL. Significant differences were observed in terms of age, first and second log(β-hCG) values, β-hCG ratio, first and second P levels, second E2 level, interday E2 level difference, and number of pregnancy losses (all P < 0.05). No significant differences were found for other characteristics. S1 Table in **S1 File** presents the baseline characteristics after excluding biochemical pregnancies. Among the 158 EPLs, 43 were classified as biochemical pregnancies and 115 as clinical pregnancy losses. Significant differences were observed in the first and second log(β-hCG) values between biochemical pregnancy and clinical pregnancy loss (S2 Table in **S1 File**).

**Table 1:** Baseline characteristics.

Characteristics	Total	Ongoing pregnancy	EPL	P-value
N	421	263 (62.47%)	158 (37.53%)	
Age, years, mean ± SD	31.22 ± 3.87	30.64 ± 3.79	32.18 ± 3.83	<0.001
BMI, kg/m^2^, mean ± SD	21.70 ± 2.87	21.50 ± 2.87	22.04 ± 2.86	0.061
First log(β-hCG), mean ± SD	2.76 ± 0.95	2.86 ± 0.97	2.60 ± 0.89	0.006
Second log(β-hCG), mean ± SD	3.12 ± 0.86	3.24 ± 0.86	2.92 ± 0.83	<0.001
β-hCG ratio, mean ± SD	2.52 ± 1.32	2.61 ± 1.33	2.37 ± 1.28	0.010
First progesterone, ng/ml, mean ± SD	32.68 ± 23.59	35.08 ± 24.36	28.69 ± 21.76	0.007
Second progesterone, ng/ml, mean ± SD	32.63 ± 23.49	35.12 ± 23.22	28.48 ± 23.41	0.005
Interday progesterone level difference, mean ± SD	−0.05 ± 15.24	0.04 ± 14.72	−0.21 ± 16.12	0.869
First estradiol, pg/ml, mean ± SD	406.38 ± 390.23	415.17 ± 426.02	391.76 ± 322.72	0.552
Second estradiol, pg/ml, mean ± SD	432.23 ± 389.62	463.08 ± 433.43	380.88 ± 297.26	0.036
Interday estradiol level difference, mean ± SD	25.84 ± 180.28	47.91 ± 185.32	−10.88 ± 165.71	0.001
Age at menarche, years, mean ± SD	13.00 ± 1.24	13.00 ± 1.20	13.01 ± 1.31	0.896
Previous miscarriage	2.02 ± 1.06	1.87 ± 0.96	2.27 ± 1.17	<0.001
Regularity of menstruation, n (%)				0.627
No	96 (22.80%)	62 (23.57%)	34 (21.52%)	
Yes	325 (77.20%)	201 (76.43%)	124 (78.48%)	

EPL, early pregnancy loss; SD, standard deviations; BMI, body mass index.

### 3.2. Correlation analysis

**Fig 2:**
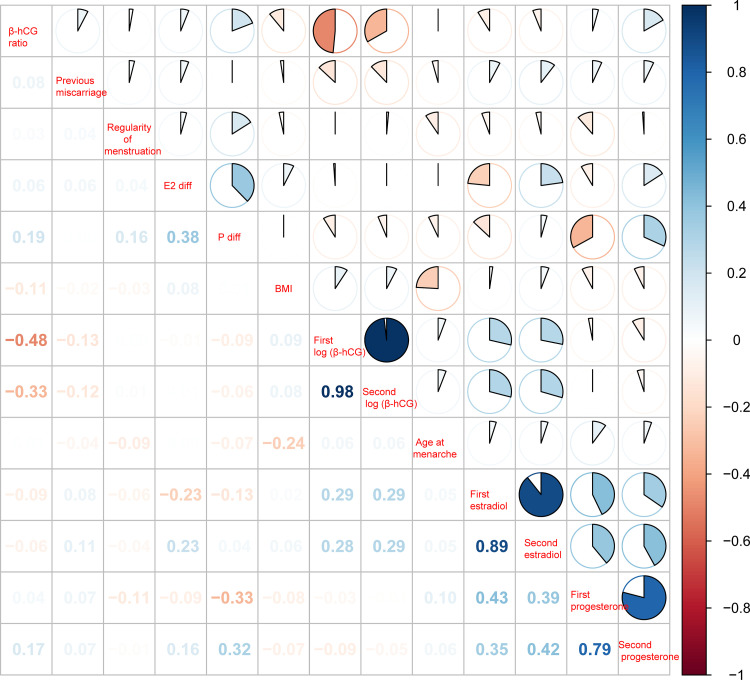
Pearson correlation coefficient.

Pearson correlation analysis was employed to examine the associations between variables. Strong correlations were observed between the repeated measurements for β-hCG (r = 0.98), P (r = 0.79), and E2 (r = 0.89) (**Fig 2**).

### 3.3. Feature selection

**Fig 3:**
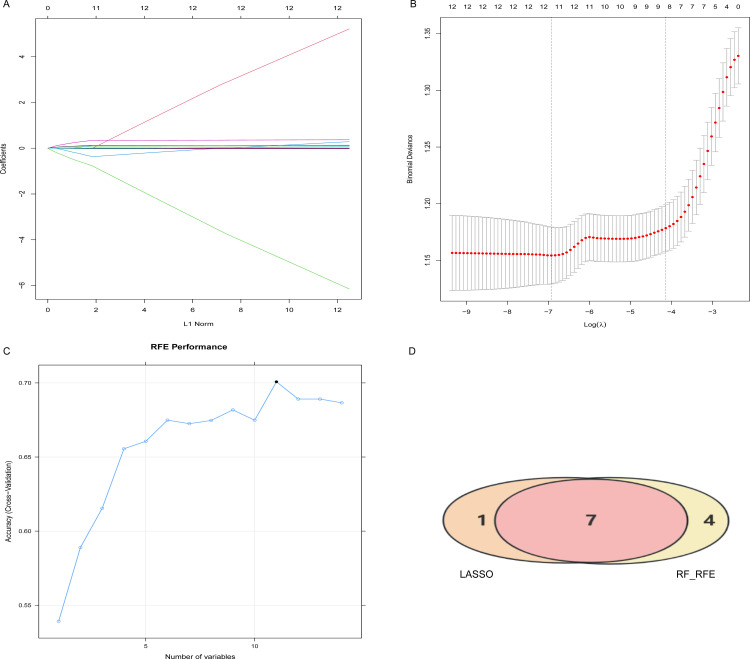
Feature screening. **(A)** Regression coefficient variation curve based on LASSO. **(B)** The optimal λ process was obtained through iterative analysis using the 10-fold cross-validation method based on LASSO. **(C)** Feature variable screening based on RF-RFE. **(D)** LASSO combination RF-RFE. LASSO: Least Absolute Contraction and Selection Operator; RF: Random Forest; RFE: Recursive Feature Elimination.

**Fig 3A** illustrates the process of variable simplification and coefficient adjustment in the LASSO regression model. It highlights the importance ranking of the variables included in the model, with each distinct colored line corresponding to a particular variable. As the penalty parameter is increased, the coefficients associated with less significant variables tend to diminish quickly, nearing zero. In contrast, the coefficients of more crucial variables exhibit minimal changes, enabling these key variables to persist throughout the modeling process until its conclusion. Meanwhile, **Fig 3B** presents the relationship between the mean squared error and the logarithm of the penalty parameter (log(λ)). This trend serves as a foundational element for determining the optimal model selection, guiding researchers in effectively balancing model complexity with predictive accuracy. The LASSO algorithm supported feature selection by regularizing the model, thereby limiting its complexity during the fitting process. Cross-validation was performed within a specified λ range, yielding two key parameters: lambda_min and lambda_lse. Lambda_min corresponded to the λ value that minimized the cross-validation error, while lambda_lse represented the largest λ within one standard error above the minimum cross-validation error. Lambda_lse was chosen to prevent overfitting and enhance model robustness. Similarly, the RF-RFE method performed feature selection by utilizing an RF-based strategy, iteratively training the model to assess the importance of each feature and recursively eliminating irrelevant features. The model achieved its highest accuracy when including 11 variables (**Fig 3C**). We ultimately selected seven common features from both the LASSO and RF-RFE algorithms to construct the predictive model, including the second β-hCG value, β-hCG ratio, age, interday E2 level difference, first P value, number of pregnancy losses, and BMI (**Fig 3D**).

### 3.4. Model construction and performance comparison

**Fig 4:**
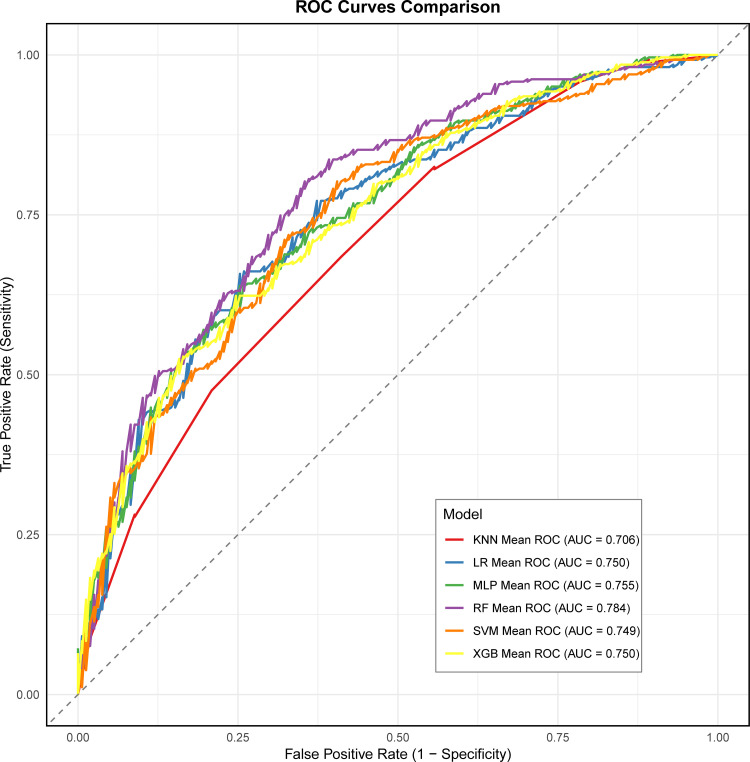
The mean ROC curves of the six models. AUC: area under the curve; LR: Logistic regression; RF: Random forest; XGBoost: eXtreme Gradient Boosting; KNN: k-nearest neighbors; SVM: Support vector machine; MLP: Multilayer perceptron.

Based on the selected features, we developed an LR model and five machine learning models. The ROC curves for all six models are presented in **Fig 4**. Among them, the RF model demonstrated the best discriminatory performance, with an AUC of 0.784, followed by the MLP (AUC = 0.755), LR (AUC = 0.750), XGBoost (AUC = 0.750), SVM (AUC = 0.749), and KNN (AUC = 0.706) (**Table 2**). The RF model ranked first in terms of accuracy and precision, while ranking third in sensitivity and second in specificity. Overall, the RF model exhibited consistently strong performance across all metrics.

**Table 2:** Performance parameters of the six prediction models.

Variable	Mean AUC (95% CI)	Accuracy (95% CI)	Precision (95% CI)	Sensitivity (95% CI)	Specificity (95% CI)
LR	0.750 (0.721,0.792)	0.703 (0.694,0.714)	0.639 (0.607,0.679)	0.480 (0.419,0.531)	0.837 (0.827,0.846)
RF	0.784 (0.757,0.796)	0.729 (0.702,0.762)	0.724 (0.647,0.773)	0.462 (0.375,0.531)	0.890 (0.887,0.904)
XGBoost	0.750 (0.725,0.795)	0.698 (0.671,0.718)	0.674 (0.654,0.667)	0.374 (0.250,0.531)	0.894 (0.887,0.923)
KNN	0.706 (0.669,0.753)	0.682 (0.647,0.714)	0.601 (0.550,0.654)	0.444 (0.344,0.531)	0.825 (0.808,0.830)
MLP	0.755 (0.751,0.761)	0.694 (0.675,0.702)	0.615 (0.579,0.615)	0.507 (0.500,0.548)	0.806 (0.750,0.849)
SVM	0.749 (0.718,0.773)	0.720 (0.702,0.729)	0.688 (0.625,0.773)	0.489(0.406,0.548)	0.859 (0.830,0.904)

AUC: area under the curve; LR: Logistic regression; RF: Random forest; XGBoost: eXtreme Gradient Boosting; KNN: k-nearest neighbors; SVM: Support vector machine; MLP: Multilayer perceptron; 95% CI: 95% confidence interval.

### 3.5. Sensitivity analysis

To assess the robustness of the findings, we excluded patients with biochemical pregnancies (n = 43) and refitted the RF model using the remaining cohort (n = 378). The model’s predictive performance slightly declined, with an AUC of 0.770 (S3 Table in **S1 File**), which may be attributed to the reduced sample size. Nevertheless, the model remained effective after excluding biochemical pregnancies, indicating overall robustness despite the minor decrease in performance.

## 4. Discussion

This study employed machine learning algorithms to integrate serum biomarkers (β-HCG, P, and E2) and their dynamic characteristics for constructing predictive models of early pregnancy outcomes. All models exhibited favorable predictive performance, with the RF model showing the optimal predictive capability. The present study is expected to provide more reliable and objective auxiliary tools for the clinical prediction of early pregnancy outcomes. The sample size can be further expanded, and more dimensions of features can be included to optimize the model’s generalization ability in the future.

Compared with the EPL group, the ongoing pregnancy group exhibited significantly higher levels of log(β-hCG) values, β-hCG ratio, P levels, second E2 level, and interday E2 level difference. Notably, even after excluding biochemical pregnancies, the ongoing pregnancy group still demonstrated elevated P levels, second E2, and interday E2 level difference relative to the EPL group. These results underscore the crucial role of hormonal levels and their dynamic fluctuations in maintaining early pregnancy. It is well-established that low levels or unfavorable trends in β-hCG, P, and E2 are widely recognized as potential indicators of adverse pregnancy outcomes. For instance, Li et al. reported that low levels and increased rates of E2 and β-hCG were associated with adverse pregnancy outcomes [11]. Deng et al. evaluated the predictive value of serum E2, P, and β-hCG before 9 weeks of gestation for predicting pregnancy loss within 12 weeks [8]. Their study identified that specific combinations of low hormone levels at different gestation ages (e.g., low E2 + P or E2 alone at 7–9 weeks, or low β-hCG or P + E2 at 5–6 weeks) could more effectively predict EPL. Additionally, Su et al. investigated the utility of weekly measurements of hCG, P, and E2 in assessing the viability of pregnancy in patients with unexplained recurrent pregnancy loss [12]. Their results similarly supported that repeated hCG and E2 measurements contribute to pregnancy risk assessment in this population. In conclusion, the values of hormone levels and their dynamic changes have significant predictive value for the outcome of early pregnancy.

Combined LASSO regression with RF-RFE, we identified seven core predictive variables: second β-hCG, β-hCG ratio, age, interday E2 level difference, first P value, number of pregnancy losses, and BMI. In line with prior investigation, dynamic changes in β-hCG demonstrated greater predictive value for pregnancy outcomes compared to a single measurement [13]. The predictive significance of age, number of previous pregnancy losses, and BMI—established risk factors for pregnancy loss—was corroborated by our findings [14]. Furthermore, P and E2 are essential hormones for early gestational maintenance [15], and their fluctuations may reflect dynamic alterations related to embryonic development or placental function. Pearson correlation analyses, performed across the entire cohort, revealed high correlations between repeated measurements of the same hormone, confirming both assay stability and data integrity. Crucially, while single measurements were highly correlated, they were not perfectly concordant. The differences between these serial measurements constitute the core of dynamic indicators. Machine learning models, particularly RF, which excels at capturing complex nonlinear relationships, effectively leveraged the information embedded within these dynamic changes to enhance predictive performance.

We assessed the efficacy of six machine learning models for predicting the risk of EPL. All models demonstrated good predictive capability, meeting the threshold criterion for high accuracy proposed by Luo et al. [16]. Among the models, the RF algorithm exhibited the optimal predictive performance, achieving the highest values for AUC, accuracy, and precision. Even after excluding biochemical pregnancies, it showed only a modest decline while maintaining high discriminatory power. This result aligns with multiple studies confirming the advantage of machine learning models in predicting pregnancy outcomes [17–19]. Specifically regarding the application of RF, research by Yehuala et al. demonstrated its superior predictive capability in estimating risk factors for pregnancy losses [20]. Our study further corroborates the effectiveness of RF in this context. It is worth noting, however, that some studies have reported other machine learning models achieving superior performance [21], indicating that the selection of the model still requires further exploration in combination with specific clinical circumstances.

The machine learning model developed in this study is intended to function as a decision-support tool in early pregnancy and reproductive medicine clinics. To facilitate practical implementation, the model requires only seven standardized inputs readily available during routine care: maternal age, BMI, number of previous pregnancy losses, and the static and dynamic values of serum β-hCG, P, and E2 (based on a 48-hour interval). At the point of care, these parameters can be entered into a simplified digital template to generate an immediate risk probability. We propose a risk-stratified management protocol based on these predictions: Low-risk patients continue with standard prenatal care; Moderate-risk patients receive intensified surveillance, including more frequent hormonal and ultrasound monitoring and personalized medical support; and High-risk patients are prioritized for specialist-led interventions, symptom-specific management for threatened miscarriage, and proactive psychological counseling. By utilizing only routine clinical data without requiring additional specialized testing, this model offers a scalable and cost-effective solution for individualized early pregnancy management.

Several limitations should be acknowledged. First, as a retrospective single-center investigation, the generalizability of the findings is limited, and the risk of inherent selection bias cannot be excluded. Prospective, multicentre studies with larger sample sizes are needed for external validation. Furthermore, the model was evaluated solely by internal cross-validation, and its performance in the external population remains to be confirmed. Optimizing thresholds based on specific clinical needs (such as high-sensitivity screening) is a crucial step for clinical translation, future applications can tailor the decision threshold based on specific clinical scenarios and further evaluate model performance under pre-set sensitivity requirements. Finally, not all potentially relevant predictors—such as maternal medical history or ultrasonographic characteristics—were incorporated, and these factors may further influence pregnancy outcomes. In future prospective, multi-center studies, we intend to systematically collect these variables to optimize the model’s comprehensiveness and generalizability.

In summary, we developed machine learning models that incorporate both static and dynamic profiles of serum biomarkers (β-hCG, P, and E2) for the prediction of early pregnancy outcomes. Among the evaluated algorithms, the RF achieved the highest predictive performance, providing promising evidence for the clinical utility of machine learning in EPL risk stratification. Despite the inherent constraints of a retrospective design, our findings underscore the value of integrating dynamic biomarkers with an appropriate machine learning algorithm. Future multicentre prospective studies and iterative model refinement will be essential to translate these results into routine clinical practice and ultimately enhance first-trimester pregnancy management.

## Supporting information

S1 File S1 Table. Baseline characteristics of patients excluded from biochemical pregnancy. S2 Table. Baseline characteristics of patients with biochemical pregnancy and clinical pregnancy loss. S3 Table. Performance parameters of the RF prediction model excluding patients with biochemical pregnancy.
(DOCX)
