# Supplementary material for: Development of machine learning models for predicting early pregnancy outcomes based on β-hCG, progesterone, and estradiol
Source: PLoS One. 2026 Apr 27;21(4):e0348114. doi: 10.1371/journal.pone.0348114 (PMC13119905; doi:10.1371/journal.pone.0348114)
Supplement: S1 File — S2 Table. Baseline characteristics of patients with biochemical pregnancy and clinical pregnancy loss. S3 Table. Performance parameters of the RF prediction model excluding patients with biochemical pregnancy. (DOCX) [file pone.0348114.s001.docx]

**Table S1.** Baseline characteristics of patients excluded from biochemical pregnancy

| Characteristics | Total | Ongoing pregnancy | Pregnancy loss | P-value |
| --- | --- | --- | --- | --- |
| N | 378 | 263 (69.58%) | 115 (30.42%) |  |
| Age, years, mean ± SD | 31.15 ± 3.82 | 30.64 ± 3.79 | 32.30 ± 3.64 | <0.001 |
| BMI, kg/m^2^, mean ± SD | 21.73 ± 2.89 | 21.50 ± 2.87 | 22.27 ± 2.87 | 0.016 |
| First log(β-hCG), mean ± SD | 2.83 ± 0.95 | 2.86 ± 0.97 | 2.75 ± 0.90 | 0.308 |
| Second log(β-hCG), mean ± SD | 3.20 ± 0.84 | 3.24 ± 0.86 | 3.09 ± 0.79 | 0.115 |
| β-hCG ratio, mean ± SD | 2.55 ± 1.25 | 2.61 ± 1.33 | 2.40 ± 1.02 | 0.117 |
| First progesterone, ng/ml, mean ± SD | 33.19 ± 23.88 | 35.08 ± 24.36 | 28.86 ± 22.24 | 0.020 |
| Second progesterone, ng/ml, mean ± SD | 33.20 ± 23.84 | 35.12 ± 23.22 | 28.79 ± 24.74 | 0.017 |
| Interday progesterone level difference, mean ± SD | 0.01 ± 14.86 | 0.04 ± 14.72 | -0.07 ± 15.23 | 0.948 |
| First estradiol, pg/ml, mean ± SD | 406.24 ± 395.01 | 415.17 ± 426.02 | 385.82 ± 313.50 | 0.507 |
| Second estradiol, pg/ml, mean ± SD | 436.02 ± 396.57 | 463.08 ± 433.43 | 374.13 ± 287.72 | 0.045 |
| Interday estradiol level difference, mean ± SD | 29.78 ± 179.29 | 47.91 ± 185.32 | -11.69 ± 157.74 | 0.003 |
| Age at menarche, years, mean ± SD | 13.00 ± 1.25 | 13.00 ± 1.20 | 13.02 ± 1.37 | 0.880 |
| Previous miscarriage | 1.99 ± 1.05 | 1.87 ± 0.96 | 2.25 ± 1.18 | 0.001 |
| Regularity of menstruation, n (%) |  |  |  | 0.697 |
| No | 87 (23.02%) | 62 (23.57%) | 25 (21.74%) |  |
| Yes | 291 (76.98%) | 201 (76.43%) | 90 (78.26%) |  |

**Table S2.** Baseline characteristics of patients with biochemical pregnancy and clinical pregnancy loss

| Characteristics | Pregnancy loss | Clinical pregnancy loss | Biochemical pregnancy | P-value |
| --- | --- | --- | --- | --- |
| N | 158 | 115 | 43 |  |
| Age, years, mean ± SD | 32.18 ± 3.83 | 32.30 ± 3.64 | 31.86 ± 4.32 | 0.518 |
| BMI, kg/m^2^, mean ± SD | 22.04 ± 2.86 | 22.27 ± 2.87 | 21.42 ± 2.77 | 0.096 |
| First log(β-hCG), mean ± SD | 2.60 ± 0.89 | 2.75 ± 0.90 | 2.19 ± 0.74 | <0.001 |
| Second log(β-hCG), mean ± SD | 2.92 ± 0.83 | 3.09 ± 0.79 | 2.45 ± 0.76 | <0.001 |
| β-hCG ratio, mean ± SD | 2.37 ± 1.28 | 2.40 ± 1.02 | 2.30 ± 1.82 | 0.695 |
| First progesterone, ng/ml, mean ± SD | 28.69 ± 21.76 | 28.86 ± 22.24 | 28.24 ± 20.66 | 0.875 |
| Second progesterone, ng/ml, mean ± SD | 28.48 ± 23.41 | 28.79 ± 24.74 | 27.65 ± 19.65 | 0.786 |
| Interday progesterone level difference, mean ± SD | -0.21 ± 16.12 | -0.07 ± 15.23 | -0.59 ± 18.49 | 0.855 |
| First estradiol, pg/ml, mean ± SD | 391.76 ± 322.72 | 385.82 ± 313.50 | 407.64 ± 349.56 | 0.707 |
| Second estradiol, pg/ml, mean ± SD | 380.88 ± 297.26 | 374.13 ± 287.72 | 398.93 ± 324.28 | 0.642 |
| Interday estradiol level difference, mean ± SD | -10.88 ± 165.71 | -11.69 ± 157.74 | -8.71 ± 187.36 | 0.920 |
| Age at menarche, years, mean ± SD | 13.01 ± 1.31 | 13.02 ± 1.37 | 13.00 ± 1.15 | 0.941 |
| Previous miscarriage | 2.27 ± 1.17 | 2.25 ± 1.18 | 2.33 ± 1.15 | 0.727 |
| Regularity of menstruation, n (%) |  |  |  | 0.912 |
| No | 34 (21.52%) | 25 (21.74%) | 9 (20.93%) |  |
| Yes | 124 (78.48%) | 90 (78.26%) | 34 (79.07%) |  |

**Table S3.** Performance parameters of the RF prediction model excluding patients with biochemical pregnancy

| Variable | Mean AUC (95% CI) | Accuracy (95% CI) | Precision (95% CI) | Sensitivity (95% CI) | Specificity (95% CI) |
| --- | --- | --- | --- | --- | --- |
| RF | 0.770 (0.761,0.807) | 0.730 (0.711,0.745) | 0.690 (0.571,0.750) | 0.217 (0.174,0.0.261) | 0.954 (0.943,0.962) |

AUC: area under the curve; RF: Random forest; 95% CI: 95% confidence interval.
